# Basal Ganglia Intraparenchymal Cyst Revealed by a Movement Disorder

**DOI:** 10.7759/cureus.101166

**Published:** 2026-01-09

**Authors:** Kaouthar Benyarou, Sanae El Hasnaoui, Samah Yousfi, Yassine Mebrouk

**Affiliations:** 1 Neurology, Mohammed VI University Hospital, Faculty of Medicine and Pharmacy, Oujda, MAR

**Keywords:** basal ganglia, cystic lesion, dystonia, magnetic resonance imaging, movement disorder, neuroepithelial cyst

## Abstract

Neuroepithelial cysts are rare, benign intracranial lesions most often detected incidentally due to their typically silent clinical course. We report the case of a 12-year-old right-handed girl who presented with progressive weakness of the left upper limb accompanied by involuntary movements of the left hemibody, including myoclonus and dystonia. Brain magnetic resonance imaging (MRI) demonstrated a well-defined intraparenchymal cystic lesion within the right basal ganglia, with cerebrospinal fluid-like signal characteristics and no contrast enhancement, radiologically suggestive of a neuroepithelial cyst. Manifestation as a movement disorder is exceedingly uncommon, particularly in the pediatric population. This case expands the clinical spectrum of basal ganglia intraparenchymal cystic lesions presenting with complex movement disorders and underscores the role of MRI in lesion characterization and clinico-radiological correlation.

## Introduction

Intracerebral cysts can be broadly categorized into neoplastic and nonneoplastic lesions. In benign nonneoplastic lesions, classification is often based on the anatomical location of the mass or the nature of the lining membrane [1,2]. Neuroepithelial cysts (NECs), also known as neuroglial cysts (NGCs), glioependymal cysts (GECs), or neuroependymal cysts, are benign developmental anomalies of the central nervous system (CNS) derived from ectodermal remnants [3]. They constitute fewer than 1% of intracranial tumors [4]. Although the histogenesis of these lesions remains unclear, most authors agree on an embryogenetic origin. The majority of these lesions are asymptomatic and are diagnosed incidentally [5]. However, some present with seizures [6,7], mass effect [8,9], or rarely movement disorders [10-12].

Despite advances in neuroimaging, establishing a definitive diagnosis of NEC based on imaging alone may be challenging, particularly for deep gray matter lesions with atypical clinical manifestations. In such cases, nonspecific radiological features and the rarity of movement disorder presentations may lead to diagnostic uncertainty or misclassification as other cystic, metabolic, infectious, or neoplastic basal ganglia disorders [13]. Moreover, detailed pediatric cases correlating the lesion location with specific movement phenomenology remain scarce in the literature. Here, we report an unusual case of an intraparenchymal cyst involving the basal ganglia and adjacent internal capsule, radiologically suggestive of a NEC, revealed by a movement disorder. This uncommon clinico-radiological correlation has been only rarely documented, emphasizing its diagnostic importance.

## Case presentation

A 12-year-old right-handed girl, with no family history of neurological disease, presented with a three-year history of gradually progressive weakness of the left upper limb associated with increasing stiffness and involuntary movements affecting the left side of the body. There was no history of infection, cerebrovascular events, head trauma, intracranial hemorrhage, neoplastic disease, surgery, or known congenital disorders.

**Figure 1:**
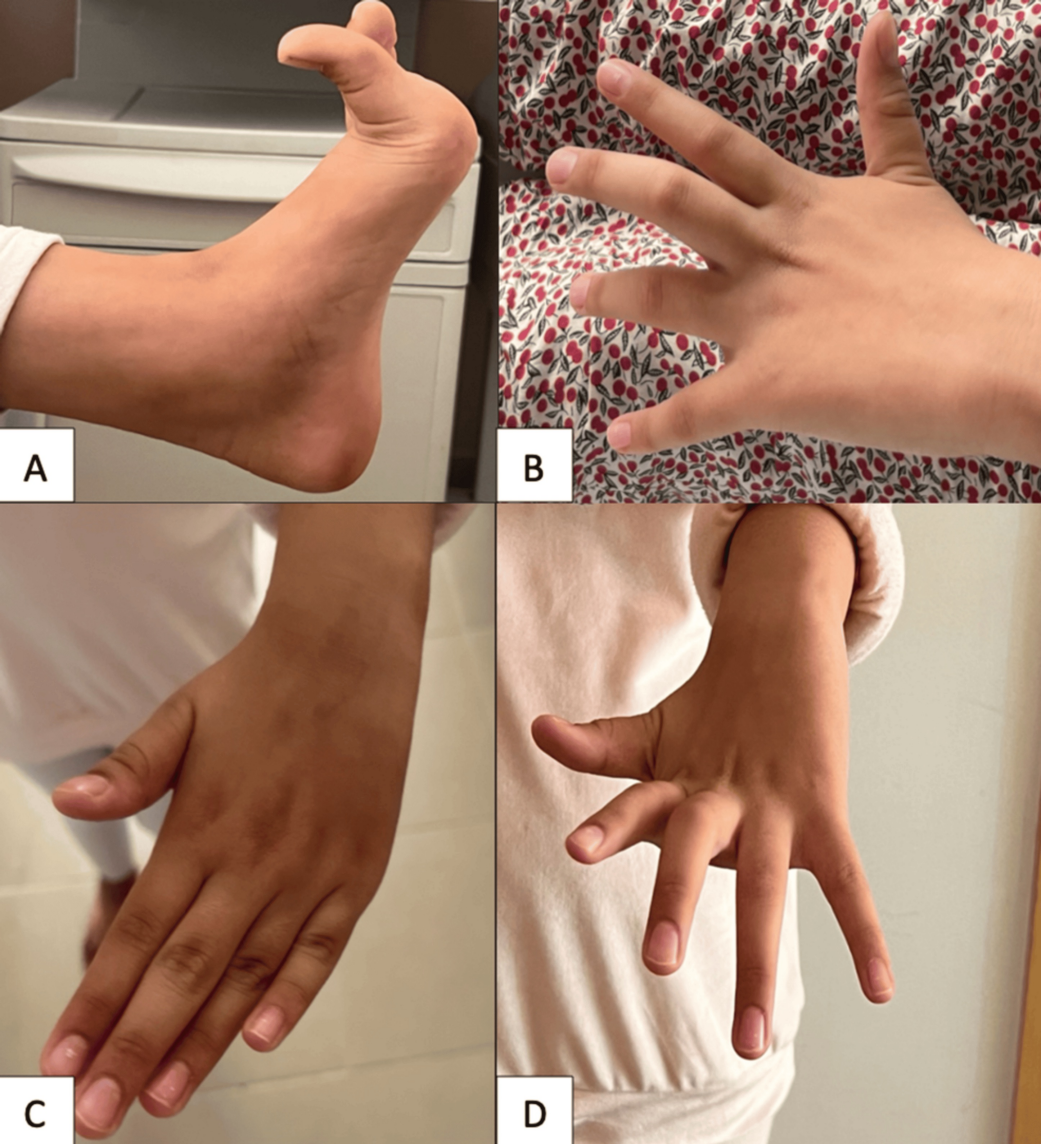
Clinical photographs demonstrating dystonic postures. (A) Forced hyperextension of the great toes. (B-D) Metacarpophalangeal hyperextension with flexion of the intermediate phalanges. (C) Forced wrist rotation.

Neurological examination revealed shock-like involuntary muscle jerks consistent with myoclonus, predominantly involving the distal segments of the left hemibody, along with sustained muscle contractions resulting in dystonic posturing. These manifestations included hyperextension of the left great toe, metacarpophalangeal hyperextension with flexion of the intermediate phalanges, and abnormal wrist rotation of the left hand (Figure 1; Videos 1, 2).

**Video 1:** Forced hyperextension of the great toe.

**Video 2:** Myoclonic jerks of the left lower limb associated with dystonic posturing of the hand.

The abnormal movements were exacerbated by stress and cognitive tasks. Muscle tone was increased in the affected limb, with uniform resistance to passive movement consistent with lead-pipe rigidity. Deep tendon reflexes were brisk bilaterally, more pronounced on the left. The remainder of the neurological examination was unremarkable.

**Figure 2:**
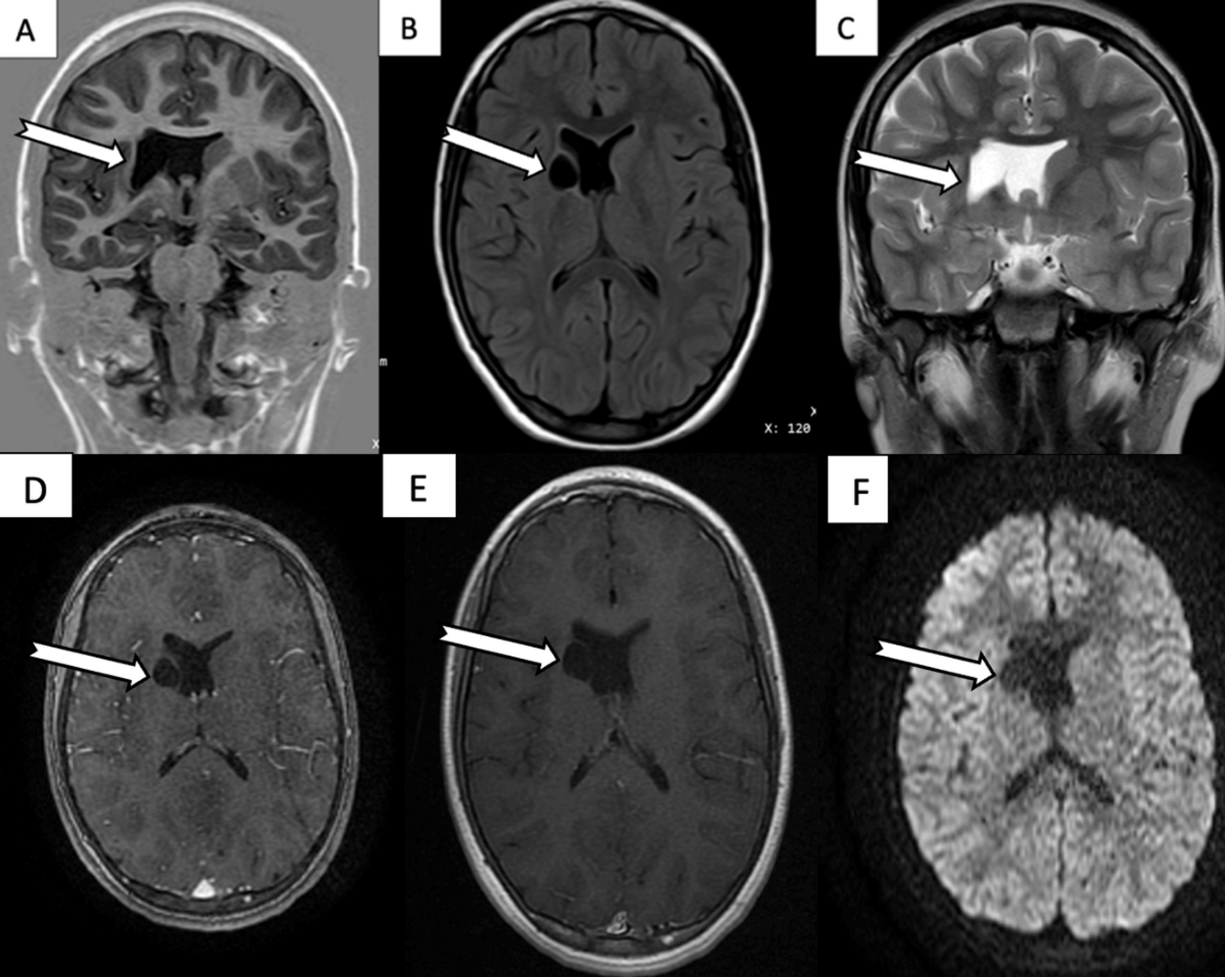
Brain MRI. (A) Coronal T1-weighted image showing a hypointense cystic lesion in the right periventricular region, adjacent to the head of the caudate nucleus and the anterior limb of the internal capsule. (B) Axial FLAIR image showing the lesion as hypointense. (C) Coronal T2-weighted image showing the lesion as hyperintense. (D, E) Axial post-contrast T1-weighted images showing no enhancement of the lesion or surrounding parenchyma. (F) DWI showing no diffusion restriction. MRI: magnetic resonance imaging; FLAIR: fluid-attenuated inversion recovery; DWI: diffusion-weighted imaging

Brain magnetic resonance imaging (MRI) revealed a well-circumscribed, round cystic lesion measuring approximately 14 × 21 mm, located in the right periventricular region adjacent to the head of the caudate nucleus and the anterior limb of the internal capsule. The lesion was hypointense on T1-weighted and fluid-attenuated inversion recovery (FLAIR) sequences and hyperintense on T2-weighted images, with a thin, regular wall and no contrast enhancement or perilesional edema. Diffusion-weighted imaging showed no signal abnormality or diffusion restriction within the lesion. Mild compression of the frontal horn of the right lateral ventricle was noted, without midline shift. These imaging features were consistent with a benign intra-axial cystic lesion, most suggestive of a NEC (Figure 2). The patient was subsequently referred for neurosurgical evaluation.

## Discussion

Historically, the terminology surrounding benign intracranial cysts has been inconsistent. Various cystic lesions may arise from trauma, stroke, congenital anomalies, tumors, or infections. Among congenital cystic masses, several histologically benign lesions exist, differing in embryological origin. Because many share overlapping clinical and radiological features, definitive diagnosis often requires histopathological examination [10,13-17].

In this clinical context, the cystic lesion was classified as intra-axial, intraparenchymal, and benign in appearance, raising several diagnostic considerations, among which a NEC represented the primary hypothesis. NECs are fluid-filled cavities lined by ependymal or epithelial cells, typically not communicating with ventricular or subarachnoid spaces. They may occur sporadically or congenitally and account for less than 0.4% of all cyst-like lesions [1,2]. NECs can be located intra-axially, intraventricularly, or in subarachnoid spaces, leading to compression of adjacent neural structures. Consequently, clinical manifestations depend on size and location, though some cysts remain asymptomatic [18].

Several embryological mechanisms have been proposed for NEC formation, including entrapment of arachnoid-derived cells, remnants of neural tube segments, sequestration of immature ependymal clusters, or migration of ependymal tissue into parenchyma after focal ventricular rupture [11]. Movement disorders are rare presentations of NECs, with tremor being most frequently reported and hemiballismus occasionally described [10,11]. These manifestations are typically associated with paraventricular cysts affecting the basal ganglia, though the pathophysiology remains unclear [19].

From a radiological perspective, NECs typically appear on CT as well-circumscribed, uniformly hypodense lesions without mural nodules or contrast enhancement [1]. On MRI, they demonstrate cerebrospinal fluid (CSF)-like signal intensity with well-defined margins and generally lack wall enhancement or perilesional edema, although septations may occasionally be present [6].

MRI findings in this patient demonstrated a deep intraparenchymal basal ganglia location, homogeneous CSF-like signal intensity across all MRI sequences, and absence of contrast enhancement, diffusion abnormalities, or surrounding edema. These features are consistent with the radiological profiles described for NEC [6]. They also allowed exclusion of other differential diagnoses, including cystic neoplastic lesions, pseudoporencephalic cysts, other congenital cysts, and infectious etiologies, owing to the absence of imaging findings suggestive of an aggressive, inflammatory, or infiltrative process, rendering these alternative diagnoses less likely [13]. Taken together, the radiological findings support the classification of the lesion as a benign intraparenchymal cyst, with a NEC representing the most plausible diagnostic hypothesis, while acknowledging the inherent limitations of an imaging-based diagnosis.

Robles et al. reported that most NECs are supratentorial (74.1%), with frontal lobe involvement most common (18.5%). Other locations include intraventricular (22.2%), interhemispheric (11.1%), infratentorial (14.8%), and rare supra-infratentorial sites (3.7%) [18]. In this patient, the basal ganglia localization of the lesion is particularly noteworthy, as it provides a plausible anatomical explanation for the observed movement disorder, possibly related to direct compression of basal ganglia structures or local monoaminergic disruption [19]. Compared with previously reported cases of NEC presenting with movement disorders-most commonly tremor or hemiballismus related to paraventricular or thalamic involvement-this presentation appears atypical in terms of both lesion location and phenomenology [10,11]. In addition to NEC, other intracranial cystic lesions may involve the basal ganglia and should be considered in the differential diagnosis. Arachnoid cysts, which represent the most common intracranial cysts, may rarely occur in the basal ganglia region and potentially lead to motor symptoms through local mass effect [19].

Given the deep intraparenchymal location of the lesion within the basal ganglia, a functionally eloquent region, invasive diagnostic or therapeutic interventions were considered to carry substantial risk in this patient. Both biopsy and surgical resection were associated with a high potential for neurological morbidity, particularly motor deficits, due to the proximity of critical basal ganglia circuits and adjacent internal capsule pathways. In the absence of radiological features suggestive of aggressive behavior, such as contrast enhancement, diffusion restriction, or progressive mass effect, a conservative management strategy was therefore favored. This decision was supported by multidisciplinary neurosurgical evaluation and was based on a careful risk-benefit assessment, emphasizing patient safety and preservation of neurological function. Close clinical and radiological follow-up was consequently adopted as the most appropriate management approach.

Management of suspected NEC depends on clinical presentation, lesion location, and symptom progression. Asymptomatic lesions are generally managed conservatively with regular clinical and radiological surveillance [19]. In symptomatic cases, surgical options may include stereotactic aspiration or endoscopic fenestration into adjacent CSF spaces [12]. More recent data suggest that endoscopic third ventriculostomy combined with cyst fenestration may represent an effective and minimally invasive option for selected deep-seated lesions, particularly in thalamic or paraventricular locations [20]. In the present case, given the intraparenchymal basal ganglia location and the potential surgical risks associated with this functionally critical region, a cautious and individualized management strategy was adopted following multidisciplinary discussion.

## Conclusions

We report a rare case of a NEC located in the basal ganglia, presenting with a combination of hemibody dystonia and myoclonus in a pediatric patient. This case highlights the importance of considering NECs in the differential diagnosis of movement disorders, particularly when symptoms are progressive and localized. The clinico-radiological correlation observed in our patient underscores the role of MRI in identifying these lesions and guiding management.

While most NECs are asymptomatic and incidentally discovered, symptomatic lesions warrant careful evaluation, with management strategies tailored to cyst size, location, and clinical presentation. Minimally invasive approaches, such as endoscopic fenestration or cyst puncture, may be considered in selected cases, particularly for deep-seated lesions, as suggested by previous reports. This report contributes to the limited literature on basal ganglia intraparenchymal cysts presenting with movement disorders and emphasizes the importance of a multidisciplinary approach to management. Although the observed movement disorder is anatomically consistent with the lesion’s location within basal ganglia circuits, a definitive causal relationship cannot be established. This case further expands the spectrum of movement disorder presentations associated with intraparenchymal cystic lesions involving the basal ganglia, highlighting atypical combinations such as dystonia and myoclonus.
